# Supplementary material for: Interpretable per case weighted ensemble method for cancer associations
Source: BMC Genomics. 2016 Jul 19;17:501. doi: 10.1186/s12864-016-2647-9 (PMC4952276; doi:10.1186/s12864-016-2647-9)
Supplement: Additional file 1 — NICK method analysis. (PDF 676 kb) [file 12864_2016_2647_MOESM1_ESM.pdf]

Interpretable per Case Weighted Ensemble Method for Cancer Associations: Supplementary material 2

Adrin Jalali, Nico Pfeifer

1 Method

SVM modified objective function [2]:

$$\min_{\mathbf{w},w_0} \left\{ \frac{1}{2}\|\mathbf{w}\|^2 + \frac{1}{2}\beta \sum_{(j,k)\in E} (w_j - w_k)^2 \right\}$$

s.t.:

$$\forall i \in \{1, \cdots, n\} : (\mathbf{w}\mathbf{x}_i + w_0)y_i \geq 1$$

Dual Problem:

$$\max_{\alpha} \left\{ \sum_{i=1}^{n} \alpha_i - \frac{1}{2} \sum_{i=1}^{n} \sum_{j=1}^{n} \alpha_i \alpha_j y_i y_j (\mathbf{x}_i^T \mathbf{L})(\mathbf{L}^T \mathbf{x}_j) \right\}$$

$$\mathbf{L}\mathbf{L}^T = (\mathbf{I} + \beta\mathbf{B})^{-1}$$

s.t.:

$$\forall i \in \{1, \cdots, n\} : \sum_{i=1}^{n} \alpha_i y_i = 0$$

$$\forall i \in \{1, \cdots, n\} : \alpha_i \geq 0$$

Laplacian matrix:

$$\mathbf{B} = \mathbf{D} - \mathbf{A}$$

Dual to Primal:
$\mathbf{w} = (\mathbf{I} + \beta\mathbf{B})^{-1} \sum_{i=1}^{n} \alpha_i y_i \mathbf{x}_i$

Our approach is summarized as:

- Reverse engineer the learned machine to extract important genes after using the network information.
- Solve SVM problem for original and transformed data.

- Calculate $\mathbf{w}$ for both models.
- Compute for each pair of nodes, for each model:
$Score(i,j) = \frac{|w_i|+|w_j|}{2} \times e^{-max(d_G(i,j),1)}$
- Report pairs with highest scores for both trained models.

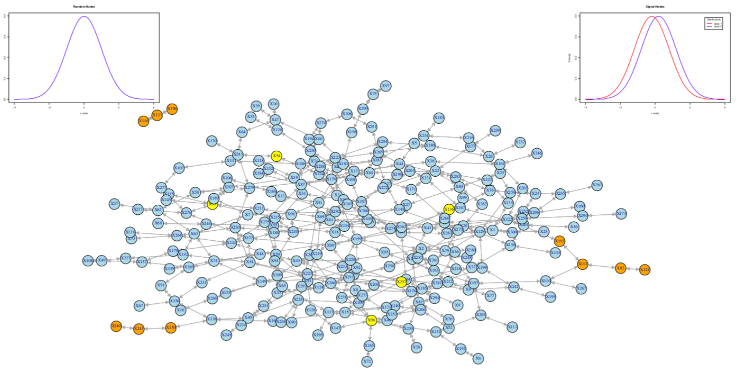

Figure 1: Blue: random gene, Orange: Signal node being a member of a pathway of signal nodes, Yellow: A lonely signal node

Fig. 1 shows an example of our random network. We select values of coresponding genes for nodes as follows:

- Signal nodes (genes): $f(n) = \begin{cases} N(-\mu, 1) & \text{if } n \text{ is in class } 1 \\ N(\mu, 1) & \text{if } n \text{ is in class } 2 \end{cases}$
- Random nodes (non-informative genes): $f(n) = N(0, 1)$

2 Results

Figures 2, 3, and 4 present the effect of the relative location of signal (predictive) nodes on the selected features. We present how a normal SVM would select features and compare that to the selected features of NICK method. We observe that although NICK performs better on the synthesized data in terms of performance measures, but it uses non-predictive features as predictive pathways move deep into the network. We observe the same effect of having the bias towards nodes that are not connected to the network on real data as well. Figure 5 presents selected features and their degree on the corresponding used graph.

3 References

1. van't Veer, Laura J., et al. "Gene expression profiling predicts clinical outcome of breast cancer." nature 415.6871 (2002): 530-536.

2. Lavi, Ofer, Gideon Dror, and Ron Shamir. ”Network-induced classification kernels for gene expression profile analysis.” Journal of Computational Biology 19.6 (2012): 694-709.

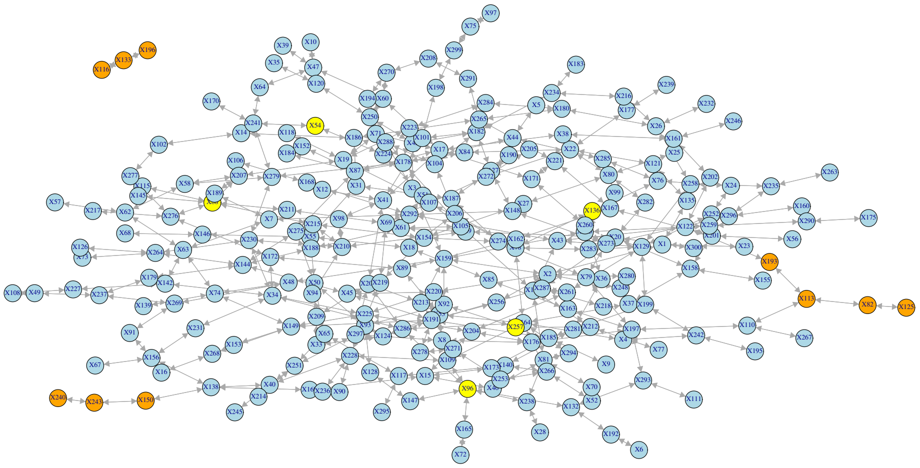

(a) Corresponding network

Original			
X196	X196	X53	X53
X233	X233	X39	X39
X88	X88	X196	X133
X116	X116	X127	X127
X197	X197	X127	X148
X148	X148	X150	X150
X148	X273	X116	X133
X160	X160	X96	X96
X95	X95	X273	X273
X88	X115	X40	X40
X53	X8	X53	X164
X195	X195	X56	X56

(b) Discovered nodes (no NICK)

Transformed			
X196	X196	X233	X233
X196	X133	X133	X133
X133	X116	X116	X116
X95	X95	X240	X240
X39	X39	X240	X243
X59	X59	X106	X106
X243	X243	X106	X168
X114	X114	X168	X168
X243	X150	X56	X56
X39	X47	X298	X298
X150	X150	X247	X247
X125	X125	X83	X83

(c) Discovered nodes (NICK)

AUC (Original):	60.6
AUC (Transformed):	62.4
wc p-value (paired):	5.669e-09

(d) Performance measures

Figure 2: An easy example: here all signal pathways are on the border of the network.

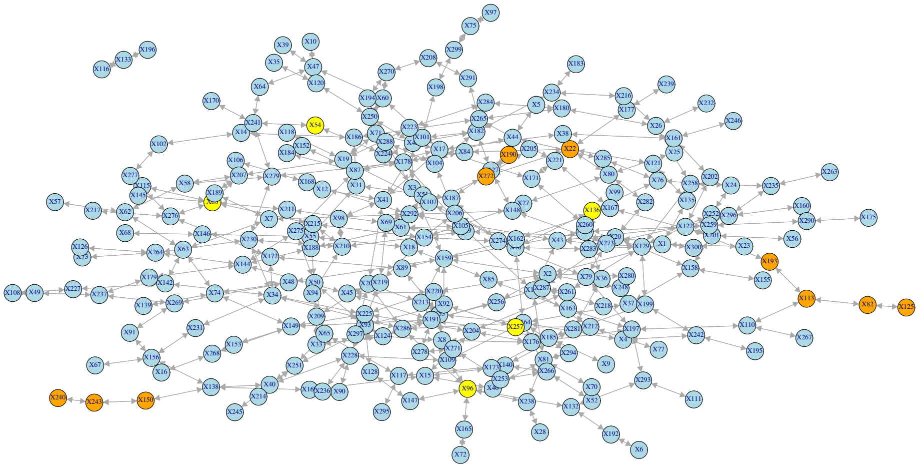

(a) Corresponding network

Original			
X190	X190	X104	X104
X233	X233	X190	X272
X277	X277	X88	X88
X190	X127	X165	X165
X272	X272	X272	X22
X106	X106	X165	X96
X150	X150	X250	X250
X88	X215	X22	X22
X51	X51	X28	X28
X73	X73	X35	X35
X162	X162	X113	X113
X112	X112	X277	X102

(b) Discovered nodes (no NICK)

Transformed			
X233	X233	X190	X190
X112	X112	X240	X240
X190	X272	X240	X243
X86	X86	X243	X243
X243	X150	X190	X127
X150	X150	X272	X272
X246	X246	X298	X298
X106	X106	X125	X125
X35	X35	X125	X82
X247	X247	X272	X69
X272	X22	X82	X82
X100	X100	X257	X257

(c) Discovered nodes (NICK)

AUC (Original):	60.1
AUC (Transformed):	61.5
wc p-value (paired):	1.383e-06

(d) Performance measures

Figure 3: A medium example: here some signal pathways are on the border of the network.

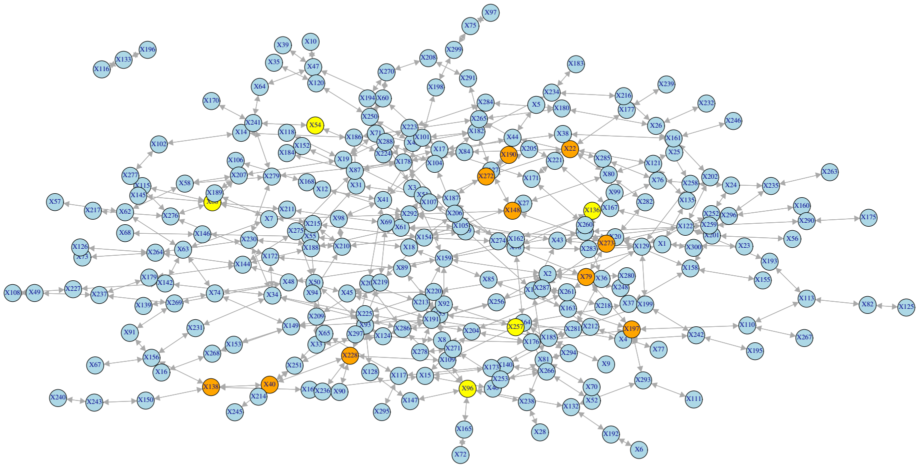

(a) Corresponding network

Original			
X190	X190	X101	X101
X233	X233	X190	X272
X88	X88	X297	X297
X190	X127	X93	X93
X26	X26	X138	X138
X272	X272	X272	X22
X101	X41	X123	X123
X22	X22	X101	X198
X146	X146	X228	X228
X278	X278	X72	X72
X88	X115	X96	X96
X148	X148	X112	X112

(b) Discovered nodes (no NICK)

Transformed			
X233	X233	X190	X190
X112	X112	X190	X272
X86	X86	X190	X127
X272	X272	X272	X205
X205	X205	X146	X146
X146	X68	X68	X68
X298	X298	X272	X22
X90	X90	X127	X127
X100	X100	X272	X69
X297	X297	X72	X72
X127	X148	X155	X155
X247	X247	X196	X196

(c) Discovered nodes (NICK)

AUC (Original):	60.2
AUC (Transformed):	62.5
wc p-value (paired):	8.151e-13

(d) Performance measures

Figure 4: A hard example: here none of the signal pathways are on the border of the network.

Original	
Node	Degree
85453	12
6605	98
56886	26
10640	16
8817	152
56894	28
5733	150
57758	8
7532	86
51	172
7566	16
3267	56
89953	4
5713	126
5193	32
5365	70
10874	132
5982	172
92140	20
332	328

(a) Discovered nodes (no NICK)

NICK	
Node	Degree
9917	0
84279	0
197370	0
51143	0
58475	0
55585	0
25949	0
54892	0
126695	0
57168	0
10456	0
148223	0
9742	0
253558	0
342527	0
10175	0
83930	0
57035	0
145482	0
57465	0

(b) Discovered nodes (NICK)

Figure 5: Comparison of selected nodes on van ’t veer data [1] using NICK and a normal SVM.
