# Supplementary material for: Interpretable per case weighted ensemble method for cancer associations
Source: BMC Genomics. 2016 Jul 19;17:501. doi: 10.1186/s12864-016-2647-9 (PMC4952276; doi:10.1186/s12864-016-2647-9)
Supplement: Additional file 2 — Detailed performance measures. (PDF 578 kb) [file 12864_2016_2647_MOESM2_ESM.pdf]

Supplementary Material 1 for: Interpretable per Case Weighted Ensemble Method for Cancer Associations

Adrin Jalali, Nico Pfeifer

1 Method's Performance

The following plots represent the performance of our method with respect to differenct regularization levels, from highly regularized to less regularized.

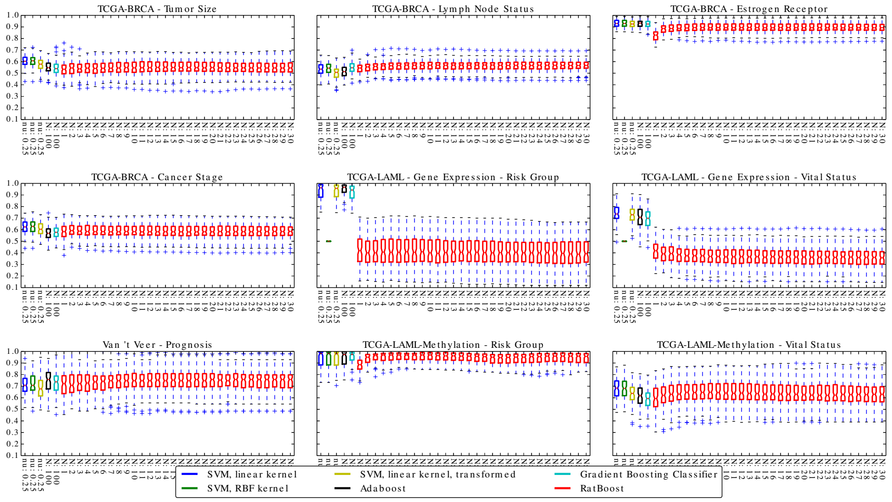

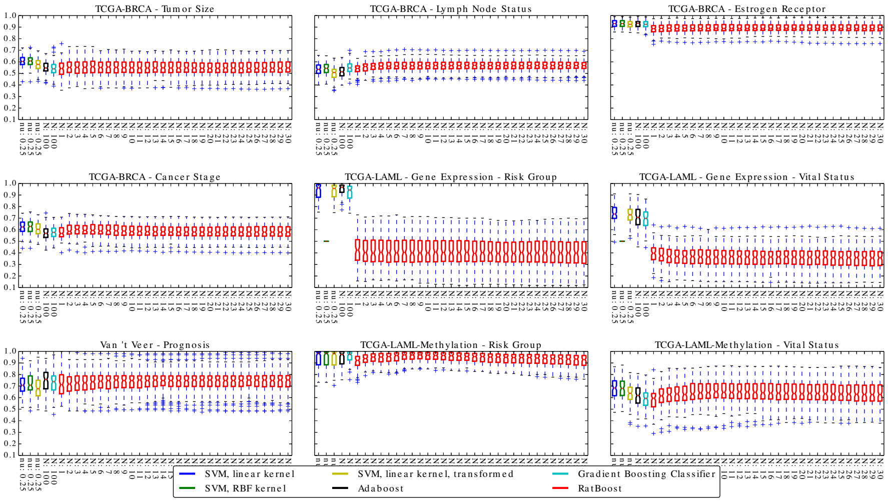
TCGA-BRCA - Tumor Size
TCGA-BRCA - Lymph Node Status
TCGA-BRCA - Estrogen Receptor
TCGA-BRCA - Cancer Stage
TCGA-LAML - Gene Expression - Risk Group
TCGA-LAML - Gene Expression - Vital Status
Van 't Veer - Prognosis
TCGA-LAML-Methylation - Risk Group
TCGA-LAML-Methylation - Vital Status
SVM, linear kernel
SVM, RBF kernel
SVM, linear kernel, transformed
Adaboost
Gradient Boosting Classifier
RatBoost

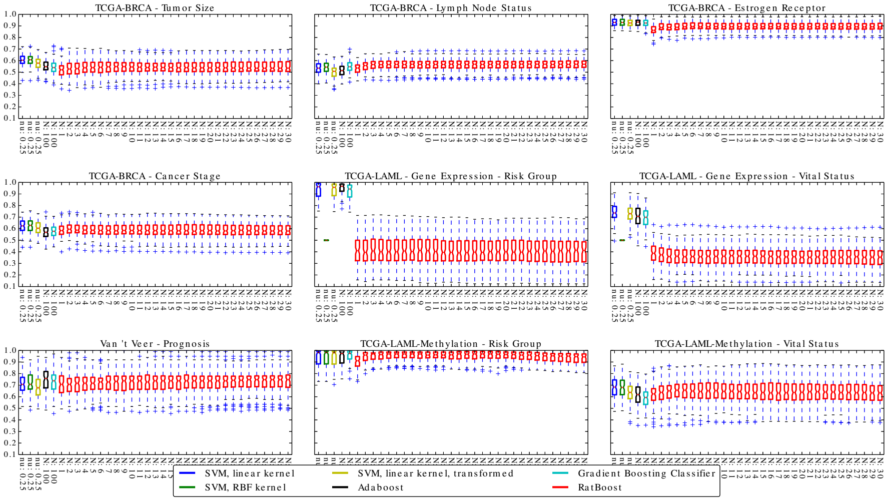
TCGA-BRCA - Tumor Size
TCGA-BRCA - Lymph Node Status
TCGA-BRCA - Estrogen Receptor
TCGA-BRCA - Cancer Stage
TCGA-LAML - Gene Expression - Risk Group
TCGA-LAML - Gene Expression - Vital Status
Van 't Veer - Prognosis
TCGA-LAML-Methylation - Risk Group
TCGA-LAML-Methylation - Vital Status
SVM, linear kernel
SVM, RBF kernel
SVM, linear kernel, transformed
Adaboost
Gradient Boosting Classifier
RatBoost

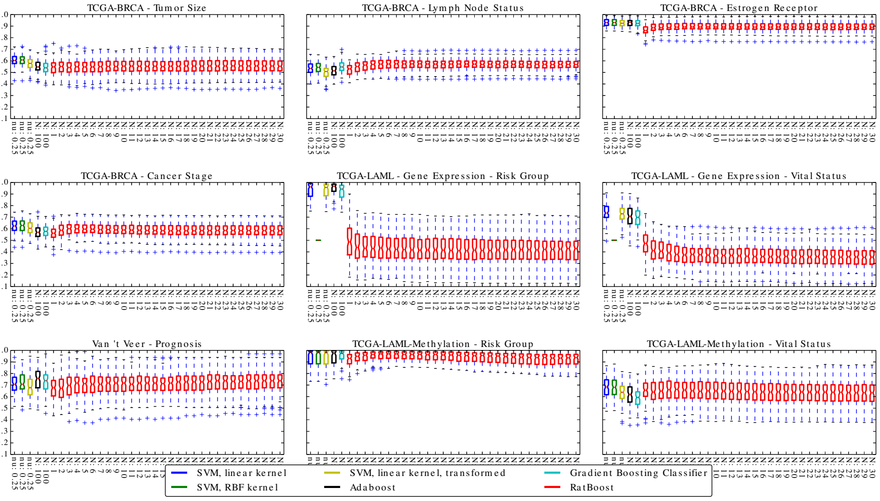
TCGA-BRCA - Tumor Size
TCGA-BRCA - Lymph Node Status
TCGA-BRCA - Estrogen Receptor
TCGA-BRCA - Cancer Stage
TCGA-LAML - Gene Expression - Risk Group
TCGA-LAML - Gene Expression - Vital Status
Van 't Veer - Prognosis
TCGA-LAML-Methylation - Risk Group
TCGA-LAML-Methylation - Vital Status
SVM, linear kernel
SVM, RBF kernel
SVM, linear kernel, transformed
Adaboost
Gradient Boosting Classifier
RatBoost

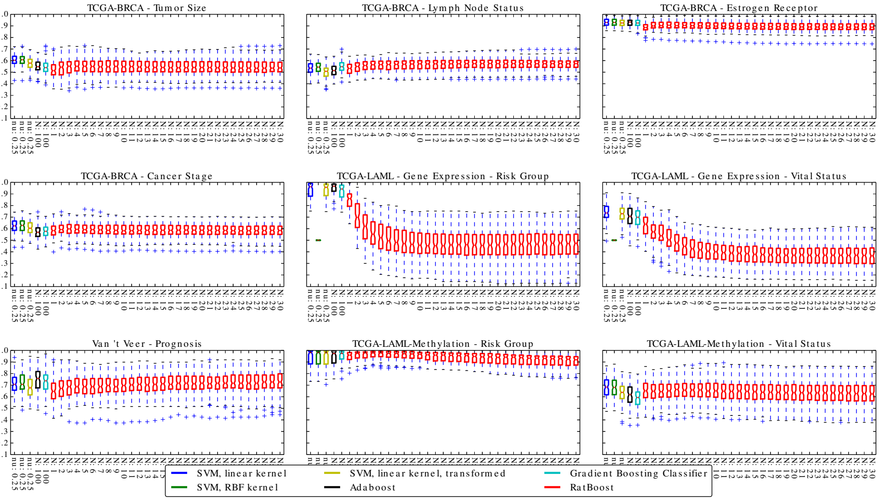
TCGA-BRCA - Tumor Size
TCGA-BRCA - Lymph Node Status
TCGA-BRCA - Estrogen Receptor
TCGA-BRCA - Cancer Stage
TCGA-LAML - Gene Expression - Risk Group
TCGA-LAML - Gene Expression - Vital Status
Van 't Veer - Prognosis
TCGA-LAML-Methylation - Risk Group
TCGA-LAML-Methylation - Vital Status
SVM, linear kernel
SVM, RBF kernel
SVM, linear kernel, transformed
Adaboost
Gradient Boosting Classifier
RatBoost

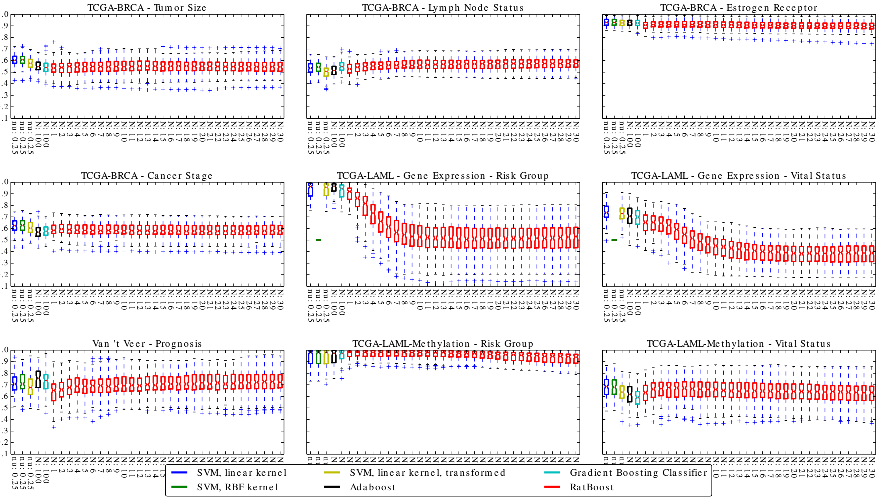
TCGA-BRCA - Tumor Size
TCGA-BRCA - Lymph Node Status
TCGA-BRCA - Estrogen Receptor
TCGA-BRCA - Cancer Stage
TCGA-LAML - Gene Expression - Risk Group
TCGA-LAML - Gene Expression - Vital Status
Van 't Veer - Prognosis
TCGA-LAML-Methylation - Risk Group
TCGA-LAML-Methylation - Vital Status
SVM, linear kernel
SVM, RBF kernel
SVM, linear kernel, transformed
Adaboost
Gradient Boosting Classifier
RatBoost

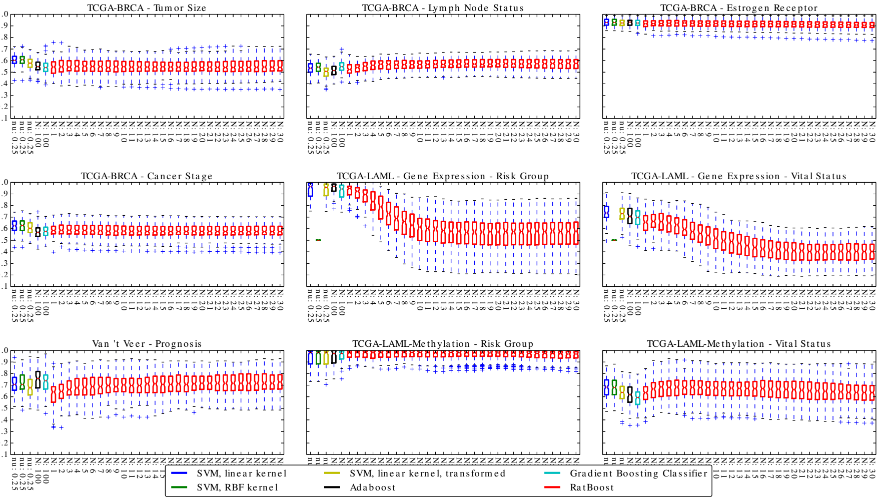
TCGA-BRCA - Tumor Size
TCGA-BRCA - Lymph Node Status
TCGA-BRCA - Estrogen Receptor
TCGA-BRCA - Cancer Stage
TCGA-LAML - Gene Expression - Risk Group
TCGA-LAML - Gene Expression - Vital Status
Van 't Veer - Prognosis
TCGA-LAML-Methylation - Risk Group
TCGA-LAML-Methylation - Vital Status
SVM, linear kernel
SVM, RBF kernel
SVM, linear kernel, transformed
Adaboost
Gradient Boosting Classifier
RatBoost

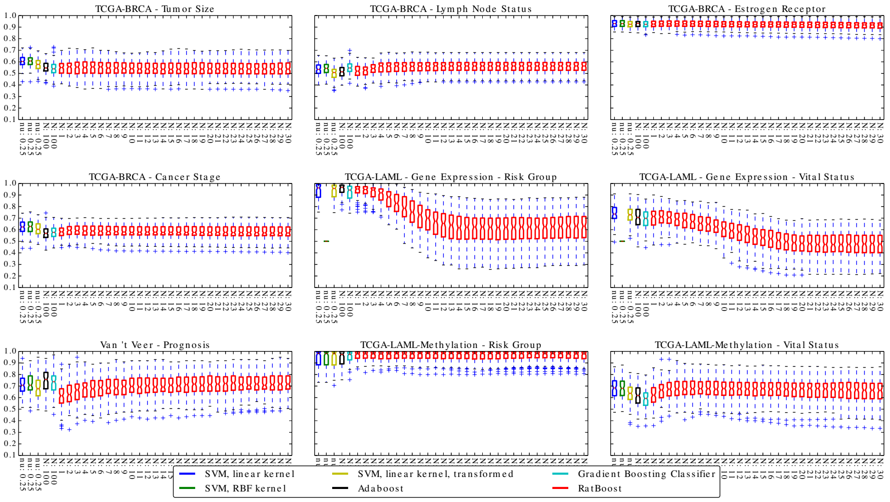
TCGA-BRCA - Tumor Size
TCGA-BRCA - Lymph Node Status
TCGA-BRCA - Estrogen Receptor
TCGA-BRCA - Cancer Stage
TCGA-LAML - Gene Expression - Risk Group
TCGA-LAML - Gene Expression - Vital Status
Van 't Veer - Prognosis
TCGA-LAML-Methylation - Risk Group
TCGA-LAML-Methylation - Vital Status
SVM, linear kernel
SVM, RBF kernel
SVM, linear kernel, transformed
Adaboost
Gradient Boosting Classifier
RatBoost

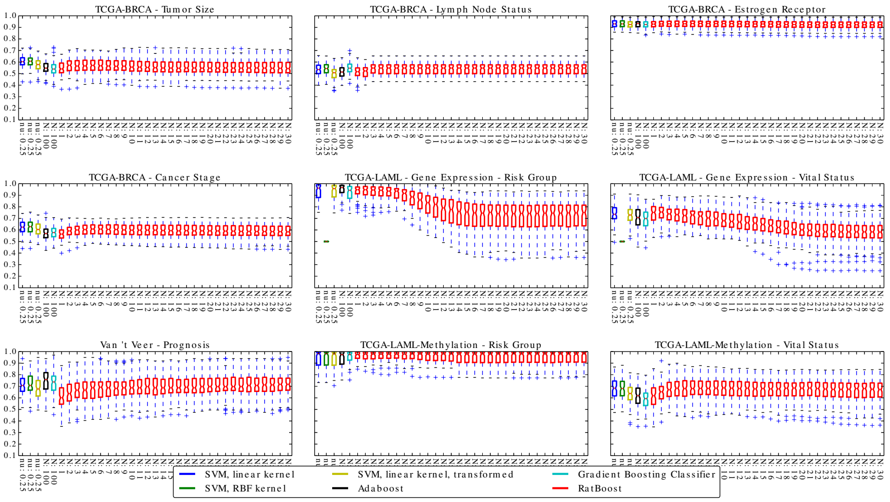
TCGA-BRCA - Tumor Size
TCGA-BRCA - Lymph Node Status
TCGA-BRCA - Estrogen Receptor
TCGA-BRCA - Cancer Stage
TCGA-LAML - Gene Expression - Risk Group
TCGA-LAML - Gene Expression - Vital Status
Van 't Veer - Prognosis
TCGA-LAML-Methylation - Risk Group
TCGA-LAML-Methylation - Vital Status
SVM, linear kernel
SVM, RBF kernel
SVM, linear kernel, transformed
Adaboost
Gradient Boosting Classifier
RatBoost
